# DEVICE-MEASURED MOVEMENT BEHAVIORS IN A NATIONALLY REPRESENTATIVE COHORT OF OLDER ENGLISH ADULTS

**DOI:** 10.1093/geroni/igae098.1175

**Published:** 2024-12-31

**Authors:** Laura Brocklebank, Aiden Doherty, Andrew Steptoe

**Affiliations:** University of Oxford, Oxford, England, United Kingdom; University of Oxford, Oxford, England, United Kingdom; University College London, London, England, United Kingdom

## Abstract

Movement behaviours impact various health outcomes. However, they have rarely been objectively measured in large nationally representative cohorts of older adults. We aim to describe the collection and analysis of device-measured movement behaviours in the English Longitudinal Study of Ageing (ELSA), and report variation by key participant characteristics. ELSA is a nationally representative cohort of English adults aged ≥50 years. In 2021-23, a random subset of 5,429 individuals were invited to wear a wrist-worn triaxial accelerometer for eight consecutive days. 4,400 agreed (81.0%) and of them 3,308 (75.2%; 55.6% men; aged 68.5 ± 9.0 years) had sufficient wear time (median [IQR] 8.0 days [7.4-8.4]). We used a machine-learning model to infer time spent in four movement behaviours (sleep, sedentary behaviour, light physical activity [LPA], and moderate-vigorous physical activity [MVPA]). Average acceleration, or overall physical activity, was 22.9 mg/day. On average, participants spent 9.4 hours/day (39.0%) sleeping, 9.8 hours/day (40.8%) being sedentary, 4.4 hours/day (18.1%) in LPA, and 30.4 minutes/day (2.1%) in MVPA. Despite men accumulating more MVPA than women, women had higher overall physical activity (23.2 vs. 22.5 mg/day) by accumulating more LPA and less sedentary time. Adults aged ≥65 years had lower overall physical activity than those aged 50-64 years (21.1 vs. 26.5 mg/day) by spending less time being active and more time being sedentary and sleeping. These data will shortly be deposited in national archives for use by other research teams and will enhance our knowledge about the potential relevance of different movement behaviours for healthy aging.

